# Causes, management outcome, and associated factors in patients admitted with a diagnosis of intestinal obstruction to Ambo University Referral Hospital: a 3-year retrospective chart review

**DOI:** 10.4314/ahs.v24i2.36

**Published:** 2024-06

**Authors:** Erko Beyene, Meti Negassa

**Affiliations:** 1 Ambo University College of Medicine and Public Health, Surgery; 2 Ambo University College of Medicine and Public Health, Internal Medicine

**Keywords:** Intestinal obstruction, management outcome, causes of intestinal obstruction

## Abstract

**Background:**

Intestinal obstruction is a major cause of surgical admissions in African countries. In this study, we assessed the causes, management outcome and associated factors among patients admitted with the diagnosis of intestinal obstruction to AURH.

**Methods:**

A retrospective chart review was conducted on all patients admitted to AURH with the diagnosis of intestinal obstruction from September 2017G.C. to August 2020G.C.

**Results:**

Sigmoid volvulus and Adhesions were the most common causes of large bowel obstruction and small bowel obstruction, respectively accounting for 85.4% and 37.3% of cases. 203(69.3%) patients were managed operatively among which 70(23.9%) had unfavorable outcome. The mortality rate of intestinal obstruction in our study population was 5.5%. Factors which had significant association with management outcome were length of hospital stay, history of abdominal surgery and history of intestinal obstruction.

**Conclusion:**

The most common causes of intestinal obstruction in our study population were similar to the one's implicated in other studies done in the low-income countries. There is relatively high rate of unfavorable outcome which calls for further investigation as to why this is occurring. We recommend also interventions to be implemented to reduce the causes of morbidity and mortality related to intestinal obstruction found in this study.

## Background

Intestinal obstruction is one of the most common surgical problems that surgeons face in their clinical practice^1^. It affects the lives of millions of individuals and is a significant cause of surgical admissions worldwide^2^. It also accounts for a great proportion of morbidity and mortality in African countries, Ethiopia being one of these^3^. Intestinal obstruction occurs when there is an interruption in the forward flow of intestinal contents. This interruption can occur at any point along the length of the gastrointestinal tract, and clinical symptoms often vary based on the level of obstruction 4.

Intestinal obstruction can be classified in many different ways. Based on the segment of the gastrointestinal tract affected, we classify it as small bowel and large bowel obstruction. It can also be either mechanical or functional on the basis of the underlying pathophysiology of obstruction.

Small bowel obstruction accounts for 80% of obstruction cases, while large bowel obstruction accounts 20% of cases^5^. Adhesions, hernias, and neoplasms are causes of small bowel obstruction in 90% of cases^6^. Of these, adhesive small bowel obstruction represents 55–75% of small bowel obstruction cases while hernias and small bowel tumors account for the remainder 7,8. Large bowel obstruction is most often the result of colorectal malignancies and the lesions usually arise in the sigmoid or recto sigmoid area 9. The other causes include volvulus, intussusception, crohn's disease, and rarely adhesions.

Although treatment options for intestinal obstruction vary considerably depending on the diagnosis, the condition of the bowel, and the status of the patient, the initial management of patients with acute intestinal obstruction should focus on an aggressive fluid replacement, decompression of the obstructed bowel, and on prevention of aspiration^3^,^10^. The next management could be surgical intervention or conservative treatment. The surgical procedures include release of adhesions, resection and anastomosis, release of constricting agents like band, de-rotation of volvulus and sigmoidoscopy^10^.

Although Surgery remains the cornerstone of treatment it could sometimes lead to a variety of postoperative complications such as incisional site infections, wound dehiscence, pneumonia, sepsis and death^11^.

Intestinal obstruction (IO) is a frequently encountered problem at the surgical emergency department responsible for frequent admissions 6. It is an important cause of morbidity and mortality in both developed and developing countries 12,13. About 3.2 million cases of bowel obstruction occurred in 2015 globally which resulted in 264,000 deaths^14^,^15^. In Africa where the majority of global cases of acute intestinal obstruction occur, the incidence was estimated to be around 12 per 100000 per year. Also, it is the region where majority of emergency abdominal surgeries are done for intestinal obstruction since it is the leading cause of acute abdomen in most of the countries^16^,^17^. Ethiopia is also one of the countries where intestinal obstruction constitutes a major cause of morbidity and mortality 3,11. According to different studies, IO roughly accounts for about 49–60% of all surgically treated acute abdominal disorder cases in Ethiopia 18,19

This study tried to find out the causes, the management outcome and factors that are associated with the management outcome among patients admitted to AURH with the diagnosis of intestinal obstruction within the study period. Though there were many studies about the causes and prevalence of intestinal obstruction in our country, only few studies were conducted on the management outcome and the factors that are associated with it. And to our knowledge there was no study done on intestinal obstruction in our study area.

Thus, this study was conducted to fill this information gap and generate base line information about the intestinal obstruction in our study area. We also believe that the results of this study give a good indication of how well our surgical services are doing and will also be an essential input for hospital administrators and other policy makers to design proper strategies to address the factors that are strongly associated with the management outcome. In addition, it will serve as a reference for those who want to undertake research on intestinal obstruction.

## Methodology

### Study Setting

The study was done in Ambo town. AURH referral hospital is a teaching hospital under AURH College of Medicine and Health Science. It has been providing service for the community since 2008E.C. It is the only referral hospital in AURH town. Surgical department is one of the 4 actively serving departments within the hospital since 2009E.C. There are 39 beds and 3 operating tables.

### Study Design and Period

Institution based retrospective chart review was conducted by reviewing patients' medical records from the past three years from September 2017 G.C- August 2020 G.C.

### Source population

All patients admitted to AURH Referral Hospital with the diagnosis of intestinal obstruction.

### Study population

All patients admitted to AURH Referral Hospital with the diagnosis of intestinal obstruction and fulfill the inclusion criteria.

### Inclusion criteria

All patients of any age who were admitted to AURH with the diagnosis of intestinal obstruction

### Exclusion criteria

Patients with incomplete records for the variables or lost records were excluded

### Sample size and Sampling Method

The calculated sample size for patients admitted to AURH with intestinal obstruction was 165. This was calculated with single population proportion formula for finite population with 95% confidence interval, 5% margin of error, prevalence of poor management outcome from similar study of 24.6%20. We also added 10% non-respondent rate to account for issues related to incomplete or lost records. Since we determined the sample size to be small to make generalized conclusions, we decided to include all patients admitted with the diagnosis of intestinal obstruction (293 Patients) to AURH from September 2017 to August 2020.

### Data collection procedures

The data was collected by reviewing patients' record. Initially, patients who were admitted with the diagnosis of intestinal obstruction were identified from logbooks in operation theatres, surgical and pediatric wards of the hospital. The medical record numbers of the patients were taken from these logbooks. Then patients' data was obtained with the medical record number and relevant information taken from the records with a structured checklist. The checklist is derived from similar studies 11,20. The checklist was developed in English language and contains information like sex, age, cause of intestinal obstruction, management mode, surgical procedure, post op complication and management outcome.

### Variables

#### Dependent variables

Management outcome of IO.

#### Independent variables

##### Socio-demographic characteristics

Age, Sex

Preoperative clinical characteristics (presenting symptoms, duration of illness—from the onset of IO symptoms up to the surgery done for IO, comorbidity, and previous abdominal surgery, Previous intestinal obstruction, Vital Signs at presentation)

Intraoperative and postoperative clinical characteristics (intraoperative diagnosis, type of intraoperative surgical procedure done, and length of hospital stay—after the surgery done until discharge from the hospital inpatient service, post-operative complications).

##### Operational Definitions

Favorable outcome if patients did not develop either postoperative complication or death after conservative or operative management of IO.

Unfavorable outcome if the patient developed one or more postoperative complications (including wound infection, facial dehiscence, anastomotic leakage, developed septic shock, pelvic collection and pneumonia), failed conservative management and/or death.

##### Data Quality control

For data collection, two Medical Doctors were recruited who were not part of the AURH Referral Hospital staff. Investigators provided training for data collectors on how to complete the checklist, as well as the importance of data quality and relevance of the study. The investigators were supervising the data collection activities, ensuring the consistency and completeness of the checklist and providing appropriate support.

##### Data Management and Analysis

Frequencies and Proportions were used for description of the study population in relation to socio-demographic and other variables. The data obtained was entered into Epi Data version 3.1 and exported to SPSS version 23 for analysis. Bivariate analysis with Binary logistics regression analysis was performed to assess the presence of crude association between independent and dependent variables. Multivariate analysis was then performed to control potential confounding variables. The strength of association between independent and dependent variables was assessed using adjusted odds ratio with 95% confidence interval and p-value of <0.05. In addition, chi square test was done to assess the association between the dependent and some of the independent variables.

### Ethical Consideration

Ethical clearance was obtained from the Research Review and Ethical Committee of AURH College of Medicine and Health sciences.

A formal letter was written to AURH from AURH College of Medicine and Health Sciences to allow and cooperate with us to find and review patient records.

## Results

### Demographic characteristics of the patients

Our study included patients from the age of 2 months up to the maximum of 89years. The mean age in the study population was 41.13years. Majority of our study population were males with male to female ratio of 3.77. (Table 1).

**Table 1:** Demographic and Clinical characteristics of study population of patients admitted to AURH with the diagnosis of IO, March 2021

Demographic characteristics		
**Sex**		
**Male**	231	79.0%
**Female**	62	21.0%
**Age**	41.13±21.73 (2months-89years)	
**Under 15**	43	14.7%
**15-65**	216	73.7%
**Over 65**	34	11.6%
**Clinical Characteristics**		
**Duration of symptoms at presentation**	3.01 ± 3.02 days	
**<=24hrs**	93	31.7%
**>24hrs**	200	68.3%
**Symptoms at presentation**		
**Abdominal Pain**	283	96.6%
**Vomiting**	248	84.6%
**Abdominal** **Distension**	246	84%
**Failure to pass faeces and flatus**	215	73.4%
**Previous intestinal obstruction**		
**Yes**	88	30.0%
**No**	205	70.0%
**Previous abdominal surgery**		
**Yes**	61	20.8%
**No**	232	79.2%
**Comorbidity**		
**yes**	56	19.1%
**no**	237	80.9%
**Vital signs at presentation**		
**Pulse Rate**	96.45±22.85 (range: 56-180)	
**SBP**	115.83±18.47 (range: 60-200)	
**DBP**	75.47±11.96 (range: 40-120)	
**Respiratory Rate**	25.71±6.67 (range: 16-68)	
**Temperature**	36.61±0.67 (range: 34-39.1)	
**Length of Hospital Stay**	8.51±7.33(range: 1-54)	

### Clinical characteristics of the patients

Our study indicated that the average duration of symptoms at presentation is 3.01days (Table 1). The most common symptom at presentation was abdominal pain accounting for 96.6% of cases. (Table 1). 30% of patients had history of previous intestinal obstruction (Table 1). The total number of patients with at least one comorbid illness were 56(19.1%) (Table 1). The most frequent comorbid illness was hypertension (40.35%). Among the study population there were 4 pregnant women. The cause of intestinal obstruction in this group were Ileosigmoid Knotting 2 (50%), Viable sigmoid volvulus 1 (25%) and Adhesive intestinal obstruction 1 (25%).

In patients aged >/= 14years there was record of blood pressure (BP) at presentation. Among this group (n=250), 226 (90.4%), 10 (4%) and 14 (5.6%) presented with normal Systolic BP (90-139mmHg), hypotension (SBP<90mmHg) and hypertension (SBP>/=140mmHg) respectively.

### Causes

The most common cause of intestinal obstruction was SBO accounting for 159 (54.3%) of admitted cases. LBO and Compound obstructions accounted for 121(41.3%) and 13 (4.4%) of cases respectively. In males, LBO was 109 (47.4%), SBO was 112 (48.7%) and Compound obstruction was 9 (3.9%) cases. In females, LBO was 12 (19.7%), SBO is 45 (73.8%) and compound obstruction was 4 (6.6%) cases. Also, the type of intestinal obstruction tends to be variable based on the age of the patients. Starting from infancy up to the age group of 35-45 the most common cause of intestinal obstruction is SBO. The incidence of LBO begins to rise in the age group of 14-25 progressively until it becomes the most common type of obstruction beginning from the age group of 45-55 years. In short, SBO dominates the earlier parts of life and LBO the later part (Table 3).

The most common cause of intestinal obstruction in our study population were found to be Sigmoid volvulus, Adhesive intestinal obstruction, and small bowel volvulus accounting for 105 (35.8%), 63 (21.5%), and 41 (14.0%) of admitted cases, respectively. Sigmoid volvulus accounted for 85.4% of cases with LBO. From these 31 (29.5%) were found to be gangrenous at presentation. The next most common cause of LBO was found to be colorectal malignancies representing 11 (8.9%) of cases. From these almost 3/4th were Rectal malignancy and the rest 1/4th was Sigmoid malignancy. Other cause of LBO, like fecal impaction (n=3), Cecal volvulus (n=1), Rectus Diathesis(n=1), Intestinal Adhesion(n=1), accounted for the rest of the cases (Table 2).

**Table 2:** Etiologies of Intestinal Obstruction in our study population, March 2021

LBO	Count	Percent
**Sigmoid Volvulus**	105	35.8*
**Viable**	74	70.5**
**Gangrenous**	31	29.5**
**Colorectal Malignancy**	11	3.8*
**Rectal cancer**	8	72.7**
**Sigmoid cancer**	3	27.3**
**Others**	7	2.4*
SBO		
**Adhesion/Band**	63	21.5*
**Viable**	55	87.3**
**Gangrenous**	8	12.7**
**Volvulus**	41	14.0*
**Viable**	35	85.4**
**Gangrenous**	6	14.6**
**Intussusception**		7.8*
**Viable**	19	82.6**
**Gangrenous**	4	17.8**
**Hernia**	21	7.2*
**Viable**	15	71.4**
**Gangrenous**	6	28.6**
**Others**	12	4.1*
Compound		
**Ileosigmoid Knotting**		3.4*
**Gangrenous**	10	100**

* ***Percent from study population***

** ***Percent from the specific etiology***

Adhesive intestinal obstruction accounted for the next most common cause of IO and most common cause of SBO representing 37.3% of cases. Among these 8 (12.7%) of were found to be gangrenous. The other common cause of SBO in our study was small bowel volvulus which accounted for 24.4% of cases. From these 6 (14.6%) were gangrenous. Hernia and Intussusception accounted for 21 (7.2%) and 23 (7.8%) of cases of intestinal obstruction, respectively. Among Hernia cases 6 (28.6%) and among intussusception cases 4 (21.5%) were found to be gangrenous (Table 2). There were also rare causes of SBO like Ileoileal knotting (n=3), Small bowel mass (n=3), Ascaris ball (n=2), Appendicoileal knotting (n=1), Mesenteric pseudocyst with adhesions (n=1), Intestinal TB (n=1).

The other cause of intestinal obstruction was Compound intestinal obstruction which was mostly due to Ileosigmoid knotting (ISK) (n=10) which accounted for 3.4% of cases of IO (Table 2). Other causes of Compound IO found in our study were adhesive intestinal obstruction (n=2) and concomitant sigmoid cancer with small bowel volvulus (n=1). All cases with ISK were found to have gangrenous bowel (either both sigmoid and ileum or only ileum). All in all, 84.6% (n=11) of cases of compound intestinal obstruction were found to have gangrenous intestinal obstruction.

Cause of intestinal obstruction across different age groups was found to be different in our study. The most common cause of intestinal obstruction in patients aged under 5 years was intussusception. After the age of 5 through the age of 35years the most common cause of intestinal obstruction was adhesive intestinal obstruction. The other common causes of intestinal obstruction in these age groups were small bowel volvulus, hernia and sigmoid volvulus. Starting from the age of 20(the minimum age recorded for sigmoid volvulus) the incidence of sigmoid volvulus rises progressively and it was the most common cause of intestinal obstruction in patients with an age greater than 35 years (Table 3).

**Table 3:** Percentage of Specific Etiology of Intestinal Obstruction in specific age group at AURH, March 2021

	Sigmoid Volvulus	Colorectal Malignancy	Adhesion/Band	Small Bowel Volvulus	Intussusception	Hernia	Ileosigmoid Knotting	Other
**<=1y**				10.0%	80.0%			10.0%
**1-5y**			33.3%	6.7%	46.7%	6.7%		6.7%
**5-14y**			55.6%	16.7%	16.7%	5.6%		5.6%
**14-25y**	8.6%	5.7%	31.4%	25.7%		11.4%	5.7%	11.5%
**25-35y**	21.9%		28.1%	18.8%	3.1%	18.8%	3.1%	6.3%
**35-45y**	32.1%	5.7%	26.4%	15.1%	3.8%	5.7%	3.8%	7.6%
**45-55y**	54.3%		10.9%	19.6%	2.2%	8.7%	4.3%	
**55-65y**	66.0%	2.0%	12.0%	6.0%		2.0%	6.0%	6.0%
**>65**	58.8%	11.8%	11.8%	5.9%	2.9%	2.9%		5.8%

**Figure 1:**
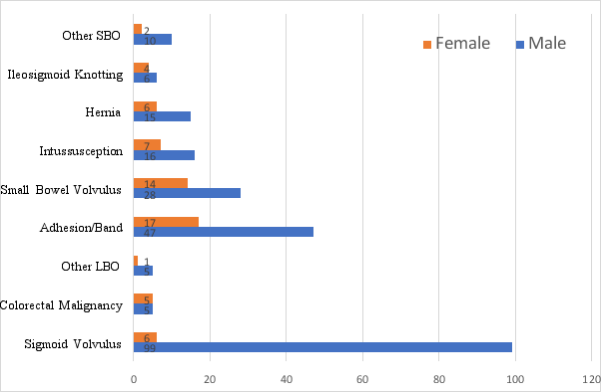
Etiology of intestinal obstruction based on sex interms of frequency in patients admitted to AURH with the diagnosis of IO, March 2021

Cause of intestinal obstruction was almost similar based on sex even though sigmoid volvulus was relatively most common in males. The most common causes of intestinal obstruction in males were successively sigmoid volvulus 99 (43%), adhesive intestinal obstruction 45(19.6%) and small bowel volvulus 27 (11.7%). Intussusception and hernia each accounted for 16 (7%) and 15 (6.5%) of cases of intestinal obstruction in males. In females, the most common cause of intestinal obstruction was adhesive intestinal obstruction 17 (27.9%), followed by small bowel volvulus 13 (21.3%) and intussusception 7 (11.5%). Hernia (n=6) and Sigmoid volvulus (n=6) each accounted for 9.8% of cases of intestinal obstruction in females (Fig 1).

Our study indicated that 64 (86.5%) of patients with viable sigmoid volvulus had history of previous intestinal obstruction. On the contrary it was only 4 (12.9%) of patients with gangrenous sigmoid volvulus who had history of previous intestinal obstruction. (Statistically significant p<0.001). Similarly, among patients with viable adhesion/band 18 (32.1%) had history of previous intestinal obstruction. In contrast, none of the patients with gangrenous adhesion/band had history of previous intestinal obstruction. Also, among patients with previous history of abdominal surgery 73.8% had adhesive intestinal obstruction.

### Management modes and related complications

**Figure 2:**
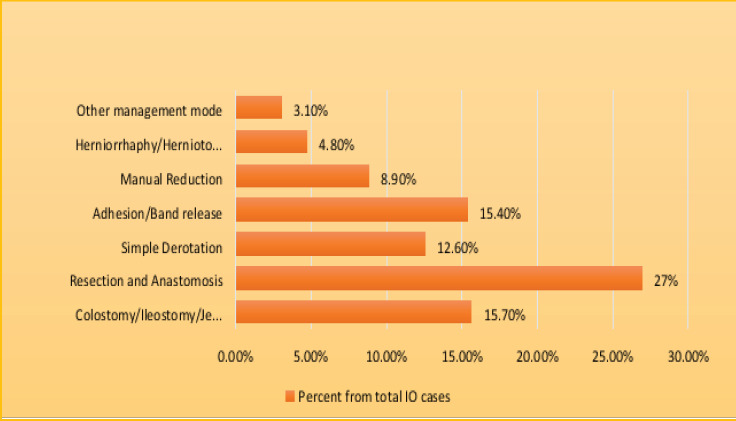
Operative procedures done interms of percentage from total study population of patients admitted to AURH with the diagnosis of IO, March 2021

Of the total of 293 patients with intestinal obstruction admitted 203 (69.3%) of them were managed with an operative procedure while the rest 90(30.7%) of them were managed conservatively. Male patients accounted for 72.6% the operated cases while the rest 26.4% of them were female. Of the patients managed conservatively 68(75.6%) were patients with LBO and 22(24.4%) of them were patients with SBO and of the operated cases 137(67.5%) were SBO. Sigmoid volvulus accounted for the majority of patients managed conservatively 65(72.2%) followed by Adhesion 15(16.7). Of the other causes of LBO, 3 patients with fecal impaction and from SBO causes, 3 intussusceptions, 2 small bowel volvulus and 1 ascaris ball cases were managed conservatively. The most common procedure performed was Resection and Anastomosis 79 (27%,) followed by Colostomy/Ileostomy/Jejunostomy (15.70%) and Adhesion/Band release (15.40%). The rest of the procedures done were simple derotation (12.60%), manual reduction (8.90%), and Herniorrhaphy/Herniotomy (4.80%) in the respective order (Fig 2).

Resection and anastomosis were majorly done for sigmoid volvulus 26(32.9%) followed by small bowel volvulus and Ileosigmoid knotting each accounting for 10(12.7%).

Colostomy/jejunostomy was also majorly done for sigmoid volvulus 26(56.5%) followed by colorectal malignancy and Ileosigmoid knotting each accounting 10(21.7%) and 5(10.9%) respectively.

**Figure 3:**
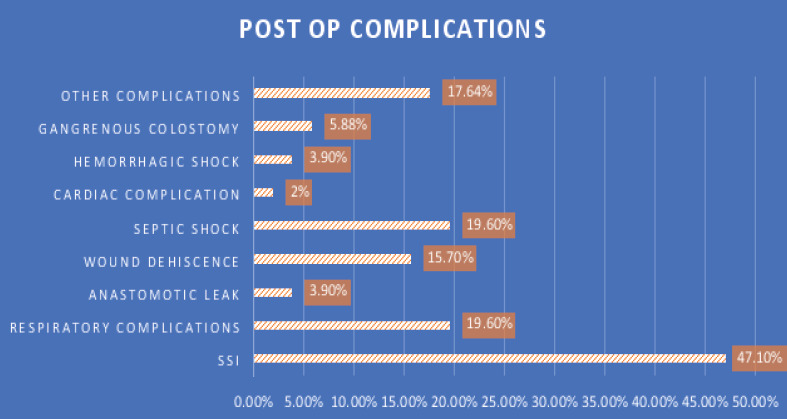
Percentage of post op complications in the operated cases from total number of complications in patients admitted to AURH with the diagnosis of IO, March 2021

Among the operated cases 51(25.12%) of the patients have developed one or more post-operative complications. The most frequently observed complication was surgical site infection accounting 24(47.10%) of patients followed by respiratory complications and septic shock, both observed in 10(19.6. %) of the patients. The other less frequently observed complications were wound dehiscence 8(15.70%), Gangrenous colostomy 3(5.88%), anastomotic leak 2(3.90%) and haemorrhagic shock 2(3.90%) (Fig 3).

Of the patients complicated with SSI, Sigmoid volvulus accounted for 33.3% followed by colorectal malignancy which was 20.8% of the cases. Adhesion/band and Ileosigmoid Knotting each accounted for 12.5% of cases with SSI. Sigmoid volvulus also accounted for the majority of the cases which complicated with respiratory complications (40.0%) followed by small bowel volvulus (30.0%).

166(56.7%) of our patients had a length of hospital stay less than or equal to 7 days. The mean length of hospital stay for our study population was found to be 8.51 with the minimum and the maximum being one day and 54 days respectively (Table 1).

### Management Outcome and Associated Factors

**Figure 4:**
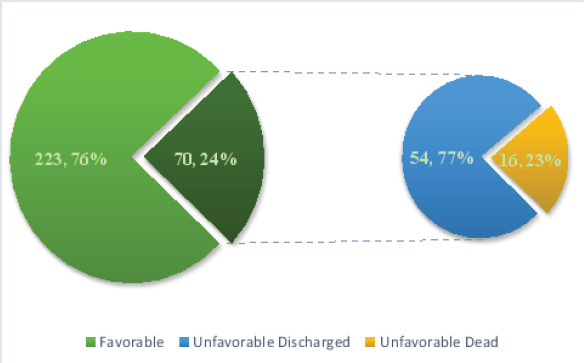
Management Outcome of patients admitted to AURH with the diagnosis of IO, March 2021

Of 293 patients with intestinal obstruction admitted to our hospital 223(76.1%) of them had a favourable outcome, whereas 70(23.9%) of the patients had unfavourable outcome. Among the patients with unfavourable outcome 54(77.1%) of them were discharged after appropriate treatment while 16 (22.9%) of the patients died making the mortality rate of intestinal obstruction in our study population 5.5% (Fig 4).

Patients with gangrenous sigmoid volvulus who had unfavorable outcome were 15(48.4%) of the cases and of these 2(6.5%) of the patients died; whereas in those with viable sigmoid patients with unfavorable outcome were only 4 (5.4%) of the cases and of this 1 patient died (statistically significant with p value<0.001). Similarly, patients with gangrenous intussusception who had an unfavorable outcome were 3 (75.0%) of cases, while the viable ones who had an unfavorable outcome were only 3(15.8%) of the cases (statistically significant p value=0.0124). Gangrenous small bowel volvulus had unfavorable outcome in 2 (33.3%) of the cases and the viable ones in 6 (16.7%) of the cases.

Of the patients with Gangrenous hernia there was only 1(16.7%) patient with unfavorable outcome as a death while no patient had an unfavorable outcome in viable hernia. Patients with rectal cancer also had a higher rate of unfavorable outcome 5 (62.5%,) of which 4(50.0%) were discharged and 1(12.5%) died due to septic shock. All cases of Ileosigmoid Knotting had a gangrenous bowel and patients had an unfavorable outcome in 60.0% of the cases. Among 10 patients admitted with the diagnosis of Ileosigmoid knotting 6 of the patients had unfavourable outcome of which 4 were discharged after the postoperative complications were managed and 2 of them died due to septic shock with possible cause of death multiple organ failure 2 to sepsis (Table 4) Comparing the mean of different variables versus the management outcome; the mean age for favourable outcome was 41.7 and for unfavorable it was 39.3. The mean for duration of symptoms at presentation was 2.91 which is lower than the mean for unfavourable outcome 3.32. The pulse rate for those who had unfavourable outcome was higher with the mean of 102.16 whereas for favourable outcome it was 94.66. The length of hospital stayas double for those with unfavourable outcome (mean= 13.76) than for those with favourable outcome (mean=6.85) (Table 5).

**Table 4:** Management outcome for specific Etiologies of Intestinal obstruction in patients admitted to AURH with the diagnosis of IO, March 2021

General Cause	Total Outcome			Total (N, %)
	Favorable (N, %)	Unfavorable Discharged (N, %)	Unfavorable Dead (N, %)	
**Sigmoid Volvulus**	86 (81.9%)	16 (15.2%)	3 (2.9%)	105 (100.0%)
**Colorectal Malignancy**	4 (40.0%)	5 (50.0%)	1 (10.0%)	10 (100.0%)
**Adhesion/Band**	43 (67.2%)	19 (29.7%)	2 (3.1%)	64 (100.0%)
**Small Bowel Volvulus**	34 (81.0%)	5 (11.9%)	3 (7.1%)	42 (100.0%)
**Intussusception**	17 (73.9%	3 (13.0%	3 (13.0%)	23 (100.0%)
**Hernia**	20 (95.2%)	0 (0.0%)	1 (4.8%)	21 (100.0%)
**Ileosigmoid Knotting**	4 (40.0%)	4 (40.0%)	2 (20.0%)	10 (100.0%)
**Total**	223 (76.1%)	54 (18.4%)	16 (5.5%)	293 (100.0%)

**Table 5:** Comparison between different study variables for the management outcomes of patients admitted to AURH with the diagnosis of IO, March 2021

Outcome		Age	Duration of symptoms at presentation	Duration of illness before surgery	PR	SBP	DBP	Length of Hospital Stay
**Favorable**	Mean	41.7	2.91	4.66	94.66	117.73	76.35	6.85
	Std. Deviation	21.7	2.96	4.52	22.56	16.72	10.91	4.56
	Maximum	89.00	30	30	180	180	110	23
	Minimum	.17	1	1	56	70	40	1
**Unfavorable**	Mean	39.3	3.32	4.71	102.16	109.84	72.70	13.76
	Std. Deviation	22.1	3.91	4.72	22.99	22.27	14.54	11.07
	Maximum	80.00	30	30	160	200	120	54
	Minimum	.25	1	1	60	60	40	1
	p-value	0.416	0.33	0.946	**0.016**	**0.003**	0.075	**0.000**

### Associated Factors

In bivariate analysis with binary logistic regression model length of hospital stay, previous history of abdominal surgery, previous history of intestinal obstruction and also the type of intestinal obstruction were associated with the management outcomes of IO in the study population at a p value of 0.25.

For these above-mentioned factors, multivariate analysis in binary logistic regression model was done and we found out that there was significant association (p<0.05) between the management outcomes of intestinal obstruction and 3 of them; these were length of hospital stay, previous history of abdominal surgery and previous history of intestinal obstruction (Table 6).

**Table 6:** Factors associated with Management outcome of intestinal obstruction at AURH, Ethiopia, March 2021

Variables		Outcome		COR (95% CI)	AOR (95% CI)	p-value
		Unfavorable	Favorable			
					
**LoHS grouped**	>7days	45	79	3.213 (1.833- 5.631)	2.879(1.594-5.201)	<0.001
	<=7days	25	141	1	1	
					
**Previous Hx of Obstruction**	Yes	12	78	0.400 (0.203-0.790)	0.303(0.137-0.671)	0.003
	No	58	147	1	1	
					
**Previous History of Laparotomy**	Yes	21	40	1.961 (1.060-3.627)	3.185(1.525-6.649)	0.002
	No	49	183	1	1	
						
**Type of Intestinal Obstruction**	SBO	37	122	1.108(0.627-1.957)	0.579(0.282-1.189)	0.137
	Compound	7	6	4.263(1.318-13.784)	2.311(0.674-7.916)	0.182
	LBO	26	95	1	1	

Patients who stayed for greater than 7 days in the hospital were about 3 times more likely to have unfavorable outcomes when compared with those who were only in the hospital for less than 7 days. (AOR=3.185, 95% CI=1.525-6.649, p=0.002) (Table 6).

Having previous history of abdominal surgery makes having unfavorable management outcome 3 times more likely. (AOR= 3.185, 95%CI=1.525-6.649, p=0.002) (Table 6).

In contrary to the above-mentioned factors, patients with previous history of intestinal obstruction were less likely to have unfavorable outcome (AOR= 0.303, 95% CI=0.137-0.671, p value=0.003) (Table 6).

Comparing patients with unfavorable and favorable outcome, pulse rate (p=0.010), SBP (p=0.003), viability of sigmoid volvulus (p<0.001), viability of intussusception (p=0.014), having colostomy/jejunostomy/ileostomy or not (p<0.001), having simple derotation or not(p=0.022), and having manual reduction or not (p=0.016) were significantly different. The patients with tachycardia and hypotension at presentation were more likely to have unfavorable outcome with odds ratios of 2.22 (1.204-4.092) and 8.021 (1.997-32.208) as compared to patients who presented with normal pulse rates and systolic blood pressures. Having gangrenous sigmoid volvulus or gangrenous intussusception at presentation were also predictive of unfavorable outcomes as compared to the ones with viable tissue (see Table 7).

**Table 7:** Factors associated with Management outcome of intestinal obstruction at AURH-II, Ethiopia, March 2021

Variables		Outcome		Relative Risk (95% CI)*	p-value
		Unfavorable	Favorable		
				
**Pulse Rate**	Normal	37 (60.7%)	154 (77.4%)	-	
	Tachycardia	24 (39.3%)	45 (22.6%)	2.22 (1.204-4.092) *	0.010
				
**SBP**	Normal	48 (78.7%)	165 (86.4%)	-	
	Hypotension	7 (11.5%)	3 (1.6%)	8.021 (1.997-32.208)	0.003
	Hypertension	6 (9.8%)	23 (12.0%)	0.897 (0.345-2.329)	0.897
				
**Viability of sigmoid volvulus**	Viable	4 (21.1%)	70 (81.4%)	-	
	Gangrenous	15 (78.9%)	16 (18.6%)	16.406 (4.798-56.096)	<0.001
					
**Viability of intussusception**	Viable	3 (50%)	16 (94.1%)	-	
	Gangrenous	3 (50%)	1 (5.9%)	16 (1.216-210.587)	0.014
					
**Presence of stoma**	Yes	27 (38.6%)	19 (12.9%)	4.237 (2.141-8.333)	<0.001
	No	43 (61.4%)	128 (87.1%)	-	
				
**Manual Reduction**	Yes	3 (4.3%)	23 (15.6%)	0.241(0.069-0.833)	0.016
	No	67 (95.7%)	124 (84.4%)		
				
**Simple derotation**	Yes	6 (8.6%)	31 (21.1%)	0.351(0.139-0.886)	0.022
	No	64 (91.4%)	116 (78.9%)		
				
				

## Discussion

### Socio-demographic characteristics

The mean age in our study was a little higher than the result obtained in some studies like Adama, Gonder and Nekemte with their mean being 32.8, 37.2, 33 respectively^11^,^20^,^21^. The same results as ours were seen in studies done in Debrebirhan, Nigeria and India 22–24. Our study also showed that the majority of patients were males as was also shown by many studies done at different parts of the world making male sex an important risk factor for developing intestinal obstruction.^5^,^11^,^20^,^22^–^25^. The possible reasons put for these especially in the African studies is that the majority of IO patients have sigmoid volvulus and it is known that sigmoid volvulus affects males in majority of cases with possible reason being the irregular bowel habits of males^22^,^24^. In contrary to this a study done in Greece showed that females accounted for 60% of patients with intestinal obstruction. In accordance to the above reasoning, this could be due to the relatively low prevalence of sigmoid volvulus in the developed world 11,22,26,27.

### Clinical presentation

Majority of our study population presented after 24 hours of symptom onset which is consistent with literatures^11^,^20^,^22^,^24^,^25^. Our study showed that the most common symptom at presentation was abdominal pain accounting for 96.6%. Comparable results were seen in many studies in the country, abdominal pain accounting for 100% in a study done in Nekemte and 95.5% in a study done in Debrebirhan^11^,^22^. Our study showed that 30% of patients had history of previous intestinal obstruction, which was much higher than a similar study done in Debrebirhan hospital in which only 8% of patients had previous intestinal obstruction^22^.

In our study 1/5^th^ of patients had history of previous abdominal surgery. This was higher when compared to studies done in different parts of the country^11^,^20^,^22^. In contrast it was less when compared with a study done in Nigeria and Turkey^12^,^24^. This disparity could be because adhesive intestinal obstruction was the most common cause of intestinal obstruction in the latter studies. In a prospective study done in Greece, all patients with adhesive intestinal obstruction had history of previous abdominal surgery. In contrary to this, our study indicated that 1/3^rd^ of the patients with adhesive intestinal obstruction did not have history of any previous abdominal operation. Some of the identified causes of intestinal adhesions in these patients were intestinal TB, Ovarian Torsion, Meckel's diverticulum and one case related to pregnancy while in the rest the cause remains unknown. Similarly, A study done in Poland implicated that bacterial infections of the peritoneum (e.g., typhoid fever, dysentery, and tuberculosis) to be the cause of adhesive intestinal obstruction in those with no prior abdominal surgery^26^. The most common indication for previous abdominal surgery in our study was Intestinal Obstruction followed by Appendicitis, Abdominal trauma and Pelvic conditions. In contrary, some other literatures showed that the most frequent prior surgical procedure performed was appendectomy^12^,^27^. And in a study done in Canada colorectal surgeries and gynecological surgeries accounted for the majority of the previous surgeries done 6.

Nearly 20% of the patients in our study had at least one comorbid illness. The most frequent comorbid illness was hypertension. Similar results were also shown in a study done in Turkey in which 21.6% had comorbid illness the most common being cardiovascular diseases^12^. Contrary to this, a study done in Gonder University hospital showed a much lower rate of comorbid illness (5.7%) in patients with intestinal obstruction^11^.

Among the study population there were 4 pregnant women. Intestinal obstruction is an infrequently encountered complication of pregnancy that is estimated to occur in approximately 1–3 of every 10,000 pregnancies^28^. However, it is the third most common nonobstetric reason for laparotomy during pregnancy (following appendicitis and biliary tract disease)^28^. The most common causes of mechanical obstruction in pregnancy are adhesions (60%) and volvulus (25%), followed by intussusception, hernia, and neoplasm^28^. The cause of intestinal obstruction in our study population were Ileosigmoid Knotting (2/4), Viable sigmoid volvulus (1/4) and Adhesive intestinal obstruction (1/4). There was also one patient who was in the postpartum period admitted with the diagnosis of adhesive intestinal obstruction.

### Etiology

Regarding the etiologies of intestinal obstruction SBO has been the commonest cause in multiple studies done in Ethiopia and different parts of the world^11^,^12^,^20^,^21^,^26^,^29^. Likewise, the most common cause of intestinal obstruction in our study was SBO accounting for more than half of the admitted cases. In contrast other studies done at Tikur Anbessa hospital and Debrebirhan showed that LBO is the most prevalent cause of the condition^22^,^29^.

In contrast to studies done in different parts of our country where the most common cause of SBO were found to be small bowel volvulus, our study indicated adhesive intestinal obstruction to be the most common cause of SBO^11^,^21^,^22^. This was similar finding with studies done at Addis Ababa, Nigeria, Turkey and Greece^12^,^24^,^27^,^29^. In our study, Sigmoid volvulus accounted for significant majority of cases with LBO followed by colorectal malignancies. Similarly, according to studies done in different parts of Africa including Ethiopia the most common cause of LBO has been sigmoid volvulus and colorectal cancer is not as prevalent as is the case in the developed world with some studies reporting absence of even a single case of malignant bowel obstruction^20^,^22^,^25^,^29^–^32^.

### Management

Intestinal obstruction is an emergency abdominal condition that requires immediate surgical intervention. Timely management decisions are very important factors in relation to morbidity and mortality. The number of patients that respond to conservative management is variable among different studies. In our study, nearly 70 % of them were managed with an operative procedure while the rest 30.0% of them were managed conservatively. According to study done in India 78.5% of patients required surgical exploration while the rest responded to conservative management^23^. Another study done in Greece showed that majority of the patients i.e., 58.7% responded to conservative management and the rest were operated (50% of which was on the first day) 32. Also, in another study done in Nigeria 57% of patients with adhesive intestinal obstruction were operated after trial of conservative management 24. Among the operated cases in our study, the most common surgical procedure performed was Resection and Anastomosis. This was similar with studies done in Adama and Gonder^11^,^20^.

The response to conservative management based on site of obstruction was also variable in different studies. In our study, LBO cases specifically sigmoid volvulus accounted for the majority of intestinal obstruction patients managed conservatively and majority of SBO cases were managed operatively. Likewise, A study done at Debrebirhan hospital also showed that majority of LBO cases i.e., 87.7% did respond to conservative management as compared to small bowel obstruction cases i.e., only 51.9% responded^22^. This may be because of large number patients with sigmoid volvulus from study area which accounted for the majority of cases of IO^22^,^32^. As opposed to these finding the study done in Greece showed that the majority of patients that responded to conservative management were the SBO group accounting for 69.3% of cases^27^.

#### Outcome

76.1% of the patients in our study had a favourable outcome, whereas 23.9% of the patients had encountered one or more post-operative complications. This result was consistent with the study done in Adama in which 24.6% of the patients with IO developed a postoperative complication^20^. Studies done in Gonder and Debrebirhan had a much lower rate of postoperative complication which was 16.7% in both studies 11,22.

According to multiple studies done the most frequently observed post op complication was surgical site infection^6^,^20^,^22^,^23^. The other frequently observed complications were wound dehiscence, septic shock and respiratory complications^6^,^11^,^20^,^22^,^23^,^29^. In the same way, our study showed that the most frequently observed complication was surgical site infection followed by respiratory complications, septic shock and wound dehiscence.

The other unfavorable management outcome was death related to IO. The mortality rate of intestinal obstruction in our study population was 5.5%, which is higher than most studies done in different parts of Ethiopia which ranged from 1.7 to 4%^11^,^21^,^22^. However, studies done in different parts of Africa showed higher mortality rates as high as 20%^24^,^25^,^31^. Study done in Greece showed mortality rate of 1.3% 27. Majority of the deaths in our study resulted from septic shock.

### Factors associated

Many studies done in our country and also abroad showed that length of hospital stay was highly associated with the management outcome^11^,^12^,^20^. Similarly, our study showed that patients who stayed for greater than 7 days were about 3 times more likely to have unfavourable outcomes. The other associated factors in our study were previous history of abdominal surgery and previous history of intestinal obstruction which were not seen in other studies. Having previous history of abdominal surgery would increase adhesion/band related comorbities. These patients would also have difficult reoperation as compared to those patients with ‘virgin’ abdomen i.e., those who have not been operated before. This is related to problems related to inadvertent bowel injuries associated with such kinds of abdomens.

In this study having history of previous intestinal obstruction was associated with increased favorable management outcome. This could be due to the majority of the patients in this study presenting with sigmoid volvulus, which is known to have recurrent behaviour, which is usually managed by simple rectal tube deflation. In addition, having previous history of intestinal obstruction would increase the awareness of the patients to the condition and augment early health seeking behaviours, ameliorating problems related with delay in health care.

In studies done in Adama, Gonder and Turkey factors like comorbidity, surgical procedures done and/or duration of symptoms before presentation were associated with management outcome 11,12,20. In our study we did not find any signicant association between management outcome and having comorbidity. However, we found association between some management procedures and the outcomes. Having colostomy/jejunostomy/ileostomy, having simple derotation, and having manual reduction were all significantly associated with management outcomes. Patients who had manual reduction or simple derotation done had significantly better/favorable outcomes as compared to the others. These procedures are likely to have better outcomes because by their nature they are not related to the breach of the wall of the intestine. In contrast patients who had stoma done post op were more likely to have unfavorable outcomes as compared to other. This could be explained by the fact that those patients who needed stoma were more likely to present with gangrenous bowel; in addition, these patients would need to stay longer hospital stays for stoma care. These factors would contribute to the poor management outcomes in these groups of patients.

## Conclusion

As recommendations, different interventions should be implemented to reduce SSI. The setup of the operation rooms, the wound care techniques and the sterilization procedures should be carefully looked at and improved. A study should also be conducted to assess the reason for the relatively high prevalence of SSI in our study population. In addition, Physicians and Nurses should all improve on chart keeping as some patient cards are incompletely filled and lack complete patient evaluation. The Card room staffs should also improve record keeping in the hospital because some charts were lost. Finally, to solve issue related to chart documentation and keeping of records we recommend the use of a unified and computerized information system in the hospital

## Abbreviations

AOR: adjusted odds ratio
CI: confidence interval
COR: crude odds ratio
IO: intestinal obstruction
LBO: large bowel obstruction
SBO: small bowel obstruction
SBV: small bowel volvulus
SD: standard deviation
SPSS: Statistical Packages for Social Science
